# Protective effects and potential mechanisms of Pien Tze Huang on cerebral chronic ischemia and hypertensive stroke

**DOI:** 10.1186/1749-8546-5-35

**Published:** 2010-10-18

**Authors:** Lihong Zhang, Wai Ping Lam, Lanhai Lü, Chunmei Wang, Yeuk Wa Wong, Lok Hang Lam, Hong Chai Tang, Maria Sen Mun Wai, Mingwei Wang, Wing Hang Kwong, Sai Ming Ngai, Ying Tat Mak, David Tai Wai Yew

**Affiliations:** 1 School of Biomedical Sciences, Faculty of Medicine, The Chinese University of Hong Kong, Shatin, Hong Kong SAR, China; 2 Department of Neurology, First Hospital of Hebei Medical University, Shijiazhuang, Hebei, China; 3 Department of Anatomy, School of Medicine, Sun Yat-sen University, Guangzhou, China; 4 Department of Biology, Faculty of Science, The Chinese University of Hong Kong, Shatin, Hong Kong SAR, China

## Abstract

**Background:**

Stroke caused by brain ischemia is the third leading cause of adult disability. Active prevention and early treatment of stroke targeting the causes and risk factors may decrease its incidence, mortality and subsequent disability. Pien Tze Huang (PZH), a Chinese medicine formula, was found to have anti-edema, anti-inflammatory and anti-thrombotic effects that can prevent brain damage. This study aims to investigate the potential mechanisms of the preventive effects of Pien Tze Huang on brain damage caused by chronic ischemia and hypertensive stroke in rats.

**Methods:**

The effects of Pien Tze Huang on brain protein expression in spontaneously hypertensive rat (SHR) and stroke prone SHR (SHRsp) were studied with 2-D gel electrophoresis and mass spectrometric analysis with a matrix-assisted laser desorption/ionization time-of-flight (MALDI-TOF)/TOF tandem mass spectrometer and on brain cell death with enzyme link immunosorbent assay (ELISA) and immunostaining.

**Results:**

Pien Tze Huang decreased cell death in hippocampus and cerebellum caused by chronic ischemia and hypertensive stroke. Immunostaining of caspase-3 results indicated that Pien Tze Huang prevents brain cells from apoptosis caused by ischemia. Brain protein expression results suggested that Pien Tze Huang downregulated QCR_2 _in the electron transfer chain of mitochondria preventing reactive oxygen species (ROS) damage and possibly subsequent cell death (caspase 3 assay) as caused by chronic ischemia or hypertensive stroke to hippocampus and cerebellum.

**Conclusion:**

Pien Tze Huang showed preventive effects on limiting the damage or injury caused by chronic ischemia and hypertensive stroke in rats. The effect of Pien Tze Huang was possibly related to prevention of cell death from apoptosis or ROS/oxidative damage in mitochondria.

## Background

Cerebral ischemia and stroke are the major causes of mortality and morbidity worldwide [1-4]. In the West, no effective treatment is available for ischemic stroke, apart from the use of aspirin and thrombolytic treatment such as tissue plasminogen activator [5]. In China, Chinese medicinal herbs have been used to treat cerebral stroke [6]. A meta-analysis of 191 trials of 22 herbal drugs found significant positive effects; however, the overall methodological quality of these trials was questionable [7,8]. The mechanisms of most herbal treatments have not been fully established [9,10].

Hypertension is the most important risk factor for ischemic stroke [1,2,11] which is often studied with animal models [12-14]. For example, the spontaneously hypertensive rat (SHR) bred from the progenitor Wistar Kyoto (WKY) rat develops hypertension spontaneously [15,16]. Stroke prone SHR (SHRsp), which has diminished cerebral arteries and thickened vessels, is characterized by 100% spontaneous hypertension and 80% cerebral stroke [17,18]. SHR and SHRsp can be used to study the effects of Chinese medicinal herbs on cerebral ischemia and hypertensive stroke respectively [17-19].

Pien Tze Huang (PZH), a herbal formula documented during the Ming Dynasty (*circa *1555 AD) in China, contains musk, *Calculus Bovis *(*Niuhuang*, ox's gallstone), *Shedan *(snake's gall) and *Panax notoginseng *(*Tianqi *or *Sanqi*) and is used to treat liver diseases, cancer and inflammation [20]. Our previous studies found that PZH protected the liver from carbon tetrachloride damage [20] and that PZH showed anti-cancer activities [21]. Our studies on Gingko suggested the possible general molecular mechanisms of neuroprotective effects [6,22,23], including apoptosis, oxidative stress, free radical in the brain cells. The components of PZH possess anti-edema, anti-inflammatory and anti-thrombotic effects [9,24,25].

The present study aims to investigate the potential mechanisms for PZH's neuroprotective effects in treating cerebral ischemia and hypertensive stroke in the SHR and SHRsp models.

## Methods

### Reagents and chemicals

All chemicals and reagents used in this study were purchased from Sigma-Aldrich (USA) or MERCK (USA) unless otherwise specified.

### Grouping and treatment of animals

Male SHR aged 3 months and age- and sex-matched control WKY rats were obtained from the Laboratory Animal Services Centre (Chinese University of Hong Kong, Hong Kong, China). Six-week old male SHRsp were obtained from the Institute of Laboratory Animal Science (Chinese Academy of Medical Sciences, Beijing, China). Housed in a room with 12 hour light-dark cycle (temperature 22-23°C and humidity 45-55%), the rats were given *ad libitum *access to standard laboratory rodent chow and water. The experiments were approved by the Animal Experimentation Ethics Committee of The Chinese University of Hong Kong.

Five groups of SHR and WKY were set up for immunostaining, proteomic analysis and cell death assay (Table 1). Briefly, Group 1: SHR for the preventive effects of PZH on ischemia (immunostaining and proteomics); Group 2: control for Group 1; Groups 3 and 4: SHR and WKY respectively for the preventive effects of PZH on ischemia (cell death assay); Group 5: SHRsp for the preventive effects of PZH on stroke.

**Table 1:** Summary of rat groups for experiments

Group	Animal	Age	Sub-group	**No**.	PZH *	Ligation	Assay	Remark
1	SHR	3 m	1A	15	Yes	Yes	I	The ischemia with PZH treatment group
			1B	15	Yes	Yes	P	The ischemia with PZH treatment group
2	SHR	3 m	2A	10	No	Yes	I	The ischemia group, control for 1A
			2B	10	No	Yes	P	The ischemia group, control for 1B
3	SHR	3 m	3A	6	Yes	Yes	C	The ischemia with PZH treatment group
			3B	6	No	Yes	C	The ischemia group, control for 3A
			3C	6	No	Sham	C	The sham operation group, control for 3B
4	WKY	3 m	4A	6	Yes	Yes	C	The ischemia with PZH treatment group
			4B	6	No	Yes	C	The ischemia group, control for 4A
			4C	6	No	Sham	C	The sham operation group, control for 4B
5	SHRsp	7 w	5A	17	Yes #	NA	C	Preventive effects of PZH in stroke
			5B	17	No	NA	C	Control group for 5A

m: month

w: week

I: immunostaining

P: Proteomic

C: Cell death detected by ELISA

* PZH was given for 3 months before the time of ligation, except for Group 5.

# For Group 5A, PZH was given until the brain stroke occurred.

Rats in Groups 1 (*n *= 30) and 2 (*n *= 20) were randomly divided into ischemia with two PZH treatments (*n *= 15) and two ischemia control (*n *= 10) groups respectively. Rats were given daily intra-gastric administration of 18 mg/kg PZH in normal saline suspension for three months before common carotid artery (CCA) ligation. Normal saline was given to rats with no PZH feeding daily.

For cell death assay, 18 SHR (Group 3) and 18 WKY (Group 4) were randomly divided into three groups (6 rats per group): (1) ischemia with PZH treatment, (2) ischemia control and (3) sham operation control. In the ischemia with PZH treatment group, before CCA ligation, rats were given daily intra-gastric administration of PZH at a dosage of 18 mg/kg for three months. The rats in ischemia control group also had intra-gastric administration of normal saline for three months before operation. The rats in the sham operation control group were not ligated.

The 34 six-week old SHRsp rats (Group 5) were given one week to adapt to the new environment. Then the rats were randomly divided into two groups: (1) 17 SHRsp rats were given 18 mg/kg PZH daily until stroke occurred and thereafter normal saline were given, (2) the rest 17 SHRsp rats were given normal saline. The rats were monitored closely everyday until signs of stroke were noted. The SHRsp rats would develop stroke spontaneously around 12-14 weeks [15,16]. Neurological status of each SHRsp was evaluated carefully by one observer who had no knowledge of the grouping. A grading scale of 0 to 4 was used to assess the extent of stroke (Table 2); scores 1 and 2 are regarded as having mild stroke and 3 and 4 as severe stroke [26]. If a rat exhibited the appropriate behavior at one step but not at the subsequent step, it was graded as the former. Neurological examinations were performed at 9:00 and 15:00 daily. Score was recorded when stroke was first noted. Once the stroke was confirmed, the feeding of PZH or saline solution would be terminated. Dates of the onset of stroke and the subsequent death were recorded.

**Table 2:** Stroke scoring

Grade	Score	Criteria
Normal	0	No observable deficit.
Mild	1	Slight reduction of activities or mild excitation.
	2	Significant reduction of activities or hyperirritability.
Severe	3	Unable to walk, decreased responsiveness.
	4	Unable to stand, limb paralysis, or paralysis of one side of the body.

The severity of stroke in stroke prone spontaneously hypertensive rats (SHRsp) was assessed with the neurologic examination grading system of stroke as previously reported [26]. When the score was 1 or 2, the severity of stroke was mild, and when the score was 3 or 4, the severity was severe.

### Ligation of common carotid arteries

Permanent occlusion of bilateral common carotid arteries (CCA) was modified from previously published methods [27,28] as follows. Bilateral CCA were ligated and excised to avoid incomplete occlusion and possible re-supply of blood flow. All equipments and tools (forceps, scissors, clamps, needles, knives, cotton, gauze and threads) were sterilized. Rats were anaesthetized by intra-peritoneal injection with 10% chloral hydrate (3 mg/kg) and placed in supine position. Hair was trimmed off the fore-neck area and skin was disinfected with 70% ethanol. A cut was made along the middle line for 1.5-2 cm and subcutaneous tissue was separated to expose the CCA located beside the thyroid gland. Distal and proximal ends of CCA were bound with 5.0 threads leaving a gap of about 0.5 cm between the two ends and the CCA was excised between the two ends. After suturing, penicillin and Temgesic (Buprenorphine) were given. Rats were returned to the cage when they could move and showed no abnormal behavior.

### Brain tissue sampling

Animal model of CCA ligation is used to study chronic ischemia effects [27,28]. Rats in Groups 1 to 4 were sacrificed two weeks following the operation, while rats in Group 5 died from stroke. For caspase-3 immunostaining, the rats of Groups 1A and 2A were anaesthetized with intra-peritoneal injection of 10% chloral hydrate (1 mg/kg). The heart was exposed after the thorax cavity was opened up. The perfusion was performed by pumping phosphate buffered saline (PBS) from the left ventricle and drained out from an opening in the right atrium. When the color of the liver turned from red to white, the perfusion continued with 300 ml of 4% paraformaldehyde (PFA) in PBS. The brain was then removed and further fixed overnight in 4% PFA. For immunostaining, different brain regions were dissected out into 5 mm long blocks which were dehydrated through a series of ethanol then cleared in xylene. The blocks were embedded and sectioned into 5 μm in coronal position for caspase-3 immunostaining.

For cell death assay and proteomic analysis, the rats of Groups 1 to 4 were decapitated and the brains were removed immediately. Tissues from hippocampus and cerebellum were sampled and after snap frozen in liquid nitrogen stored at -80°C until analysis. Upon the death of the rats of Group 5, the brains were similarly removed and hippocampus and cerebellum were dissected out and stored at -80°C until analyses for cell death index with enzyme link immunosorbent assay (ELISA) kits (Roche Diagnostics, Germany). The hippocampus was chosen as this is the area of the mid-brain area most susceptible to ischemia induced by the CCA ligation, while the blood supply to the posterior part of the brain such as cerebellum would be less affected because the vertebral arteries were intact [29].

### Immunostaining for cleaved caspase-3

Immunostaining was performed according to our previously published method [30] with primary antibody for caspase-3. All solutions for the immunostaining were diluted with 1× PBS unless otherwise stated. The tissue sections were first immersed in 0.1% Triton X-100 for 10 minutes, rinsed with PBS, incubated with 3% hydrogen peroxide (H_2_O_2_) in absolute methanol for 30 minutes and then rinsed again. Bovine serum albumin of 0.1% was used as non-specific antigen blocking solution for one hour, the sections were incubated with diluted H Capase-3 (1:500) (9662, Cell Signaling Technology, USA) overnight at 4°C. In the following day the sections were rinsed with PBS and incubated in diluted anti-rabbit secondary antibody (1:1,000) for two hours. The sections were then rinsed and incubated in diluted streptavidin horseradish peroxidase conjugated solution (1:200; Zymed^® ^Laboratories, USA) for two hours. Tissue sections for negative control were processed similarly but without primary antibody added. After the primary and secondary antibody reactions, the sections were subsequently reacted with Vectastain ABC-Peroxidase kit (Vector Laboratories, USA) for visualization of the positive cells according to our previously reported method [30]. Briefly, three consecutive slides were used for counting with a grid of 100 small squares in a 1.0 × 9 × 1.0 mm^2 ^was put into the eyepiece. Four areas measuring each 0.4 × 0.4 mm^2 ^were counted in each slide. Selection of the grid area was automatic and random by the Neurolucida 2000 software version 4.10d (MBF Bioscience, USA).

### Cell death by ELISA

Cell death detection ELISA (Roche Diagnostics, Germany) assays were carried out according to the manufacturer's procedures for detecting histone-associated DNA fragments or nucleosomes that were released from DNA degradation during apoptosis. The kit can be used for cell culture as well as different tissue homogenate samples including frozen brain tissue [31-34]. The cytosolic fraction was prepared from brain tissue sample homogenized in 0.5 ml of lysis buffer, i.e. 50 mM Tris-HCl (pH7.4) containing 150 mM NaCl, 5 mM EDTA, 0.1% sodium dodecyl sulphate (SDS), 1% Triton X-100, 0.1 mg/ml PMSF, 1 mg/ml leupeptin, 1 mg/ml pepstatin A and 5 μg/ml aprotinin. The homogenate was centrifuged at 14,000 rpm (Eppendorf, Germany) for 30 minutes at 4°C and the supernatant obtained was either immediately used or stored at -80°C until analysis. The protein concentration of the extract was determined with the protein assay (BioRad Laboratories, USA). For the ELISA, 100 μl of anti-histone antibody solution was added into each well of the microplate, then covered with the adhesive cover foil and incubated for one hour at ambient temperature (15-25°C). After incubation, the antibody solution was replaced by 200 μl of incubation buffer for a further incubation of 30 minutes. The wells were then washed three times with 250 μl of washing solution. Samples (100 μl) were added into the wells for incubation of 90 minutes. For the blank readings, incubation buffer was added instead of the samples. After incubation, wells were washed three times with 250 μl of washing solution followed by 100 μl of conjugate solution containing anti-DNA-peroxidase into each well except the blanks. After another incubation of 90 minutes, the wells were washed three times with 250 μl of washing solution followed by 100 μl of substrate solution. The plate was read at 405 nm against the blanks. Results were expressed as absorbance at 405 nm per mg of protein.

### Proteomic analysis

Comparative proteomic techniques were used to determine the preventive effect of PZH on the brain [35-37]. Hippocampus and cerebellum samples (stored at -80°C) from the ischemia with PZH treatment (Group 1B) and ischemia control (Group 2B) groups were prepared in lysis buffer (7 M urea, 2 M thiourea, 0.01% TBP, 4% CHAP and 0.01% NP 40) with protease inhibitors (GE Healthcare Life Sciences, Sweden). After total protein concentration determined with PlusOne™ 2D Quant kit (GE Healthcare Life Sciences, Sweden), equal amounts of samples of the same group were pooled into one single sample. Therefore there were four samples from two brain regions of each group. The four samples were run in triplicates (total 12 gels) and proteins were resolved with two-dimensional (2D) gel electrophoresis and silver staining. Differentially expressed spots were selected for mass spectrometry (MS) identification with a matrix-assisted laser desorption/ionization time-of-flight (MALDI-TOF)/TOF tandem MS as described in our previous publications [9,38].

#### Two-dimensional gel electrophoresis

All experiments of 2D gel electrophoresis were performed following the modified protocol of GE Healthcare Life Sciences (Sweden). Briefly, samples (150 μg) were loaded onto immobilized pH strips. Isoelectric focusing was performed on Ettan™ IPGphor™ Isoelectric Focusing System (GE Healthcare Life Sciences, Sweden) with a total voltage-hour (Vh) of 57730 progressively increased from low to high voltage (30 V, 150 V, 500 V, 1000 V to 3500 V). After equilibration in buffer (50 mM Tris-HCl, 6 M urea, 30% v/v glycerol, 2% w/v SDS, 0.002% w/v bromophenol blue) with 100 mg/10 ml DTT for 15 minutes, and then with 250 mg/10 ml iodoacetamide in the same buffer for another 15 minutes, the second dimension was separated by 11% SDS-PAGE gel at 75 V for 16 hours. After silver staining (PlusOne™ Silver Staining kit, GE Healthcare Life Sciences, Sweden), gels were scanned with an Image Scanner (GE Healthcare Life Sciences, Sweden). Image analysis was performed with ImageMaster™ 2D Platinum V.5.0 software (GE Healthcare Life Sciences, Sweden). The spots of differentially expressed proteins (fold difference ≥2) were excised from the gels and the ischemia with PZH treatment (1B) and ischemia control (2B) groups were compared [39]. The excised gel pieces were destained and digested with 8-10 μl modified sequencing grade trypsin (20 ng/μl) (Promega, USA) at 30°C for 14-16 hours. Tryptic peptides were extracted with sonication from gel pieces with 2.5% trifluoroacetic acid in 50% acetonitrile for 10 minutes.

#### Protein identification with mass spectrometry

Proteins were identified with a MALDI-TOF/TOF tandem mass spectrometer ABI 4700 proteomics analyzer (Applied Biosystems, USA). For acquisition of mass spectra, 0.5 μl samples were spotted onto a MALDI plate, followed by 0.5 μl matrix solution (4 mg/ml α-cyano-4-hydroxycinnamic acid in 35% acetonitrile and 1% trifluoroacetic acid). Mass data acquisitions were piloted by 4000 Series Explorer™ Software (version 3.0, Applied Biosystems, CA, USA) with batched-processing and automatic switching between MS and MS/MS modes. All MS survey scans were acquired over the mass range 700-3500 m/z in the reflectron positive-ion mode. The MS spectra were internally calibrated with porcine trypsin autolytic products (m/z 842.509, m/z 1045.564, m/z 1940.935 and m/z 2211.104). The MS peaks (MH+) were detected on minimum S/N ratio≥20 and cluster area S/N threshold≥25 without smoothing and raw spectrum filtering. The filtered precursor ions with a user-defined threshold (S/N ratio≥50) were selected for the MS/MS scan. Fragmentation of precursor ions was performed with MS-MS 1 kV positive mode with collision-induced dissociation on and argon as the collision gas. MS/MS spectra were accumulated from 3000 laser shots with default calibration with Glu-Fibrinopeptide B from 4700 Calibration Mixture (Applied Biosystems, USA). The MS/MS peaks were detected on minimum S/N ratio≥3 and cluster area S/N threshold≥15 with smoothing.

The MS and MS/MS data were loaded into the GPS Explorer™ software (version 3.5, Applied Biosystems, USA) and searched against NCBInr protein database by Mascot search engine (version 1.9.05, Matrix science, UK) with combined MS (peptide-mass-fingerprint approach) with MS/MS (DeNovo sequencing approach) analysis for protein identification. Search parameters were as follows: monoisotopic peptide mass (MH+); 700-3500 Da; one missed cleavage per peptide; enzyme, trypsin; taxonomy, Mus; pI, 0-14; precursor-ion mass tolerance, 50 ppm; MS/MS fragment-ion mass tolerance, 0.1 Da; variable modifications, oxidation for methionine. Top ten hits for each protein search were reported. Proteins with MOWSE score greater than 70 and at least four matched peptides were accepted as identified.

#### Functional annotation

Functional annotation and clustering were performed for the differentially expressed proteins as identified by MS with software available from two web sites, namely Database for Annotation, Visualization and Integrated Discovery (DAVID) [40,41], and Gene Ontology Tree Machine (GOTM) [42]. DAVID and GOTM are tools for data analysis and visualization for sets of genes or their products, i.e. proteins, using the Gene Ontology (GO) and the Gene Ontology Consortium 2000 which is a structured and precisely defined vocabulary for describing the roles of genes and their products with respect to biological process, molecular function and cellular component of the genes' products. In the analysis, the background list for rat (*Rattus norvegicus*) was selected. Subsets of proteins were clustered according to the functional annotations of the differentially expressed protein lists. When the enrichment score was greater than 1.0 and *P *value was less than 0.05 for a cluster, the clustering of these proteins was regarded as significant. Individual protein in the significant clusters with *P *value (with multiple testing corrections) of less than 0.05 was regarded as the protein functionally significant for the term of the cluster and the protein.

### Statistical Analysis

All data were randomized during recording and were later presented as mean ± standard deviation (SD) after grouping. Student's t-test was performed to evaluate the differences between groups for cell death assay and cleaved caspase-3 immunostaining results between the two SHRsp groups. For the cell death assay results of the three SHR and WKY groups, analysis of variance **(**ANOVA) was used to test the statistical significance of the differences among groups and *post hoc *test (with Bonferroni correction for multiple comparisons) was used to compare individual pairs of groups. The stroke scores of the SHRsp groups were analyzed with Chi-square test and the means of the time of survival after stroke of the SHRsp groups were analyzed with Student's t-test. A difference was considered statistically significant when *P *value was less than 0.05.

## Results

### Cell death assay

**Figure 1:**
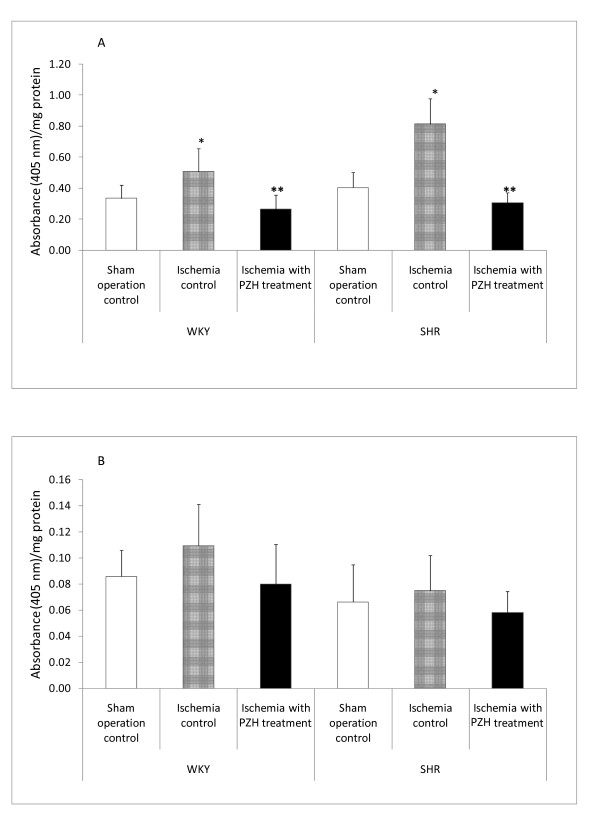
**Cell death assay results in SHR and WKY**. Cell death assay results in (1A) hippocampus and (1B) cerebellum samples from sham operation, ischemia and ischemia with PZH treatment groups of WKY and SHR two weeks after the ligation operation. For hippocampus (1A), in both WKY and SHR, ischemia significantly increased the cell death as compared to the sham operation groups (* *P *< 0.05). PZH protected hippocampus from the damage induced by ischemia and showed significant decreases (**P < 0.05) as compared to the ischemia groups. For cerebeullum (1B) there was no significant difference between the cell death result of the WKY and SHR groups.

We evaluated the cell deaths in hippocampus in the SHR (i.e. Groups 3A, 3B and 3C), WKY (i.e. Groups 4A, 4B and 4C) and SHRsp (i.e. Groups 5A and 5B) rats after ligation (with and without PZH treatment) or sham operation. The WKY (F = 7.55, P = 0.005) and SHR (F = 32.22, P < 0.001) groups showed significant differences in hippocampus and the *post hoc* tests showed that the ischemia WKY and SHR groups had significantly higher cell deaths (0.508 ± 0.148 and 0.814 ± 0.162 respectively) than the WKY and SHR sham operation groups (0.336 ± 0.085 and 0.405 ± 0.099 respectively) (WKY: P = 0.017; SHR: P < 0.001) (Figure 1A). Importantly, cell deaths in the ischemia with PZH treatment WKY and SHR groups (0.265 ± 0.085 and 0.305 ± 0.066) showed no significant difference as compared to their respective sham operation groups (Figure 1A), suggesting that PZH significantly decreased the cell death resulted from cerebral ischemia in hippocampus in both hypertensive and normotensive rats, SHR and WKY respectively. In cerebellum there was no significant difference between the sham operation control, ischemia control and PZH treatment groups (Figure 1B).

**Figure 2:**
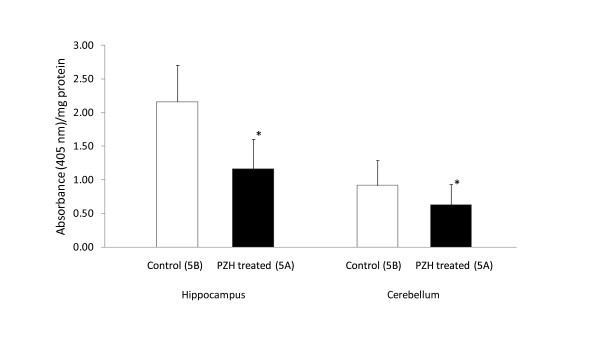
**Cell death assay results in SHRsp**. Cell death in hippocampus and cerebellum samples from SHR-sp. Group 5A was fed with daily PZH until the stroke occurred, while Group 5B was given saline. Hippocampus and cerebellum tissues were sampled when the rats died after stroke. SHRsp treated with PZH (Group 5A)  before stroke showed significantly less (* P < 0.05) cell death as compared to those rats without PZH treatment (Group 5B) in both hippocampus and cerebellum.

The hippocampus and cerebellum samples obtained from the SHRsp showed that there were significantly less cell deaths in the PZH treated group (Group 5A) when compared to the control (Group 5B) in the hippocampus (Group 5A: 1.17 ± 0.44; Group 5B: 2.16 ± 0.54; P < 0.001) and cerebellum (0.63 ± 0.30; 0.92 ± 0.37; P = 0.018) (Figure 2), suggesting that PZH preventive treatment played a significant protective role against cell deaths caused by stroke in the hippocampus and cerebellum.

### SHRsp stroke and scores

Initially, there were 17 SHRsp rats each in Groups 5A and 5B, but three rats died in Group 5A shortly after the experiment started. Using the scores from the grading system of stroke, we compared the severity of stroke between Group 5A, rats with PZH treatment before stroke and Group 5B, controls with no PZH treatment. When SHRsp were categorized by initial stroke scores, 8 out of 14 (57%) of PZH treatment rats had mild scores of 1 or 2 compared to 11 out of 17 (65%) of the control SHRsp that had severe scores of 3 or 4, although this trend was not statistically significant (Table 3). Furthermore, group PZH treated SHRsp did not show significant differences in the means of scores, time of onset of stroke and time of death compared with the control rats (Table 4). Nevertheless, one significant difference noted was the time interval between the onset of stroke and death. PZH showed significant delay (P < 0.001) of death of PZH treated rats after stroke had occurred when compared to the control rats by 2.36 ± 1.45 and 0.94 ± 0.56 days respectively (Table 4). In other words, PZH significantly lengthened the time of survival after stroke. Our results suggest that PZH treatment before the onset of stroke significantly lengthens the survival of the animals after stroke.

**Table 3:** Neurological scores at the onset of stroke

	Number of SHRsp						
Score #	1	2	3	4	Mild (1 and 2)	Severe (3 and 4)	Total
PZH treated (5A)*	1	7	2	4	8 (57%)	6 (43%)	14
Control (5B)	0	6	5	6	6 (35%)	11 (65%)	17

# score when stroke occurred

* Chi-square = 2.5 (*P *= 0.476) as compared with controls using individual scores of 1, 2, 3 and 4.

**Table 4:** (B) Score, time of onset of stroke, and time of death of the PTH treated (5A) and control (5B) SHRsp

Group	Value	Score	Stroke (day)	Death (day)	Death since stroke (day)
PZH treated (5A)	Mean	2.64	52.71	55.07	2.36
	SD	1.01	7.58	8.27	1.45
Control (5B)	Mean	3.00	54.12	55.06	0.94
	SD	0.87	10.33	9.94	0.56
	p value **	0.2973	0.6757	0.9970	0.000837

** comparing between PZH treated and control groups

### Immunostaining of caspase-3

**Figure 3:**
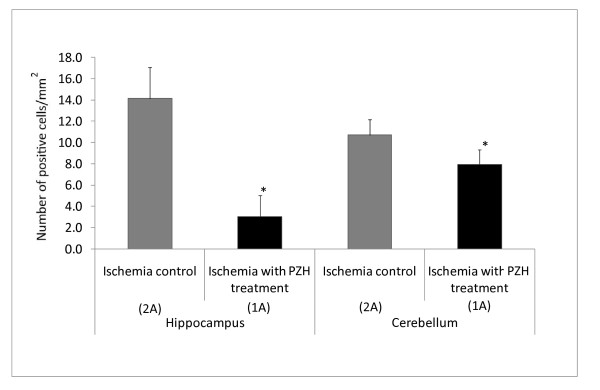
**Immunostaining results of SHR**. Cleaved caspase-3 expressions in hippocampus and cerebellum of Groups 1A and 2A PZH treatment significantly decreased apoptosis by lowering of the active cleaved caspase-3 numbers in both cerebellum and hippocampus (*P < 0.001).

**Figure 4:**
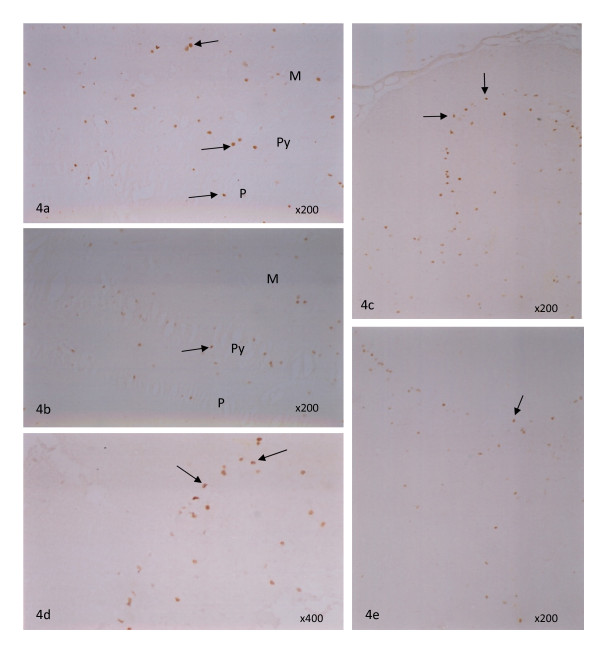
**Microscopic images of immunostaining**. Representative microscopic photos of cleaved caspase-3 positive staining cells in hippocampus (a, b) and cerebellum (c, d, e). Caspase-3 immunostaining of hippocampus samples from (a) ischemia control group 2A and (b) ischemia with PZH treatment group 1A. (a) Caspase-3 positive cells (arrows) were seen at the molecular (M), pyramidal (Py) and polymorphic (P) layers of hippocampus from a rat of ischemia control group, ×200. (b) Few caspase-3 positive cells (arrow) were found mainly in the pyramidal layer of hippocampus from a rat of ischemia with PZH treatment group, ×200. Caspase-3 immunostaining of cerebellum samples from (c, d) ischemia control group 2A and ischemia with (e) PZH treatment group 1A. (c) Caspase-3 positive cells were seen at the Purkinje layer (arrows) of cerebellum from a rat of ischemia control group, ×200. (d) A larger magnification of (c) to show the caspase-3 positive cells (arrows), ×400. (e) Only isolated caspase-3 positive cells (arrow) were found along the Purkinje layer of cerebellum from a rat of ischemia with PZH treatment group, ×200.

Cleaved caspase-3 (the activated form) expression by immunostaining was significantly lower in hippocampus in the ischemia with PZH treatment group (1A) compared to the ischemia control group (2A) (3.06 ± 1.97; 14.1 ± 2.93 cells/mm^2^; P < 0.001) and in cerebellum (7.97 ± 1.38; 10.7 ± 1.44; P < 0.001) (Figure 3), suggesting that PZH may significantly prevent apoptosis in these two brain areas as caused by the chronic ischemia. Representative photos of cleaved caspase-3 positive staining in hippocampus and cerebellum are shown in Figure 4.

### Proteomic analysis

**Figure 5:**
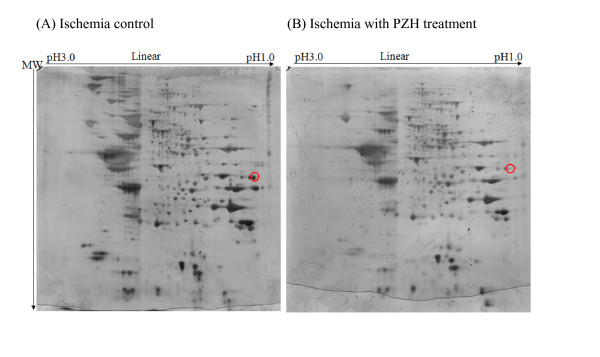
**Representative images of 2D electrophoresis gels**. Representative 2D electrophoresis gel photos of brain tissue samples from (A) SHR of ischemia with PZH preventive treatment and (B) SHR of ischemia control. The red circle indicates cytochrome b-c1 complex subunit 2, mitochondrial precursor (QCR2), the protein that down-regulated in both hippocampus and cerebellum in PZH treatment group as compared to controls. There was also no overall inhibition or over expression of proteins in the PZH treatment sample compared with the control sample.

There were nine and 13 differentially expressed proteins identified for hippocampus and cerebellum respectively (Tables 5 and 6) between the ischemia with PZH treatment group (1B) and the ischemia control group (2B). Cytochrome b-c1 complex subunit 2 (QCR2) was down-regulated in both the hippocampus and cerebellum. Both DAVID and GOTM analyses revealed that the most significant association for the hippocampus and cerebellum was with mitochondrial membrane (Table 7). Full clustering results from DAVID and GOTM for the hippocampus (Additional file 1) and cerebellum (Additional file 2) and the directed acyclic graphs (DAG) of the clustering results from GOTM for hippocampus (Additional file 3) and cerebellum (Additional file 4) are provided as additional files. Representative gel images showed that there were no other apparent inhibitions or over expressions of proteins in various samples from the two groups except those mentioned above (Figure 5).

**Table 5:** Lists of differentially expressed proteins identified by 2D-gel electrophoresis and mass spectrometric analysis using a matrix-assisted laser desorption/ionization time-of-flight (MALDI-TOF)/TOF tandem mass spectrometer of hippocampus

Swiss-Prot Ac	Refseq Protein	Protein ID	Fold *	Protein Score	Protein Name
Q99MZ8	NP_116002	LASP1	-2.80	251	LIM and SH3 domain protein 1
P20788	NP_001008888	UCRI	-2.67	448	Cytochrome b-c1 complex subunit Rieske, mitochondrial precursor
P09117	NP_036629	ALDOC	-2.53	197	Fructose-bisphosphate aldolase C; Brain-type aldolase
P54311	NP_112249	GBB1	-2.53	170	Guanine nucleotide-binding protein G(I)/G(S)/G(T) subunit beta-1
P32551	NP_001006971	QCR2	-2.40	165	Cytochrome b-c1 complex subunit 2, mitochondrial precursor
P62260	NP_113791	1433E	-2.40	296	14-3-3 protein epsilon; Mitochondrial import stimulation factor L subunit
Q5XIH3	NP_001006973	NDUV1	-2.00	236	NADH dehydrogenase [ubiquinone] flavoprotein 1, mitochondrial precursor
P63039	NP_071565	CH60	+2.63	595	60 kDa heat shock protein, mitochondrial precursor
P08461	NP_112287	ODP2	+3.20	178	Dihydrolipoyllysine-residue acetyltransferase component of pyruvate dehydrogenase complex, mitochondrial precursor

* - denotes down-regulated and + denotes up-regulated comparing the ischemia with PZH group (1B) to the ischemia control group (2B).

**Table 6:** Lists of differentially expressed proteins identified by 2D-gel electrophoresis and mass spectrometric analysis with a matrix-assisted laser desorption/ionization time-of-flight (MALDI-TOF)/TOF tandem mass spectrometer of cerebellum samples

Swiss-Prot Ac	Refseq Protein	Protein ID	Fold *	Protein score	Protein Name
P15999	NP_075581	ATPA	-5.60	460	ATP synthase subunit alpha, mitochondrial precursor
P32551	NP_001006971	QCR2	-5.07	99	Cytochrome b-c1 complex subunit 2, mitochondrial precursor
P00507	NP_037309	AATM	-4.17	446	Aspartate aminotransferase, mitochondrial precursor
P01026	NP_058690	CO3	-2.80	146	Complement C3 precursor
Q9JHU0	NP_075412	DPYL5	-2.93	412	Dihydropyrimidinase-related protein 5
O08651	NP_113808	SERA	-2.67	67	D-3-phosphoglycerate dehydrogenase
P67779	NP_114039	PHB	-2.13	561	Prohibitin
P10860	NP_036702	DHE3	-2.03	443	Glutamate dehydrogenase 1, mitochondrial precursor
Q63270	NP_059017	ACOC	+2.00	154	Cytoplasmic aconitate hydratase

* - denotes down-regulated and + denotes up-regulated comparing the ischemia with PZH group (1B) to the ischemia control group (2B).

**Table 7:** The most significant cell component group of functional annotation by DAVID and GOTM of the differentially expressed proteins of the hippocampus and cerebellum by 2D gel electrophoresis and mass spectrometric analysis with a matrix-assisted laser desorption/ionization time-of-flight (MALDI-TOF)/TOF tandem mass spectrometer

Method	Brain region	Term	*P *value	Benjamini *	Proteins
DAVID	Hippocampus	Mitochondrion	0.0000185	0.01497	NP_036629, NP_112287 *, NP_071565 *, NP_001006973, NP_001006971, NP_001008888.
	Cerebellum	Mitochondrial inner membrane	0.0000317	0.02553	NP_075581, NP_001006971, NP_036702, NP_114039, NP_037309.
GOTM	Hippocampus	Mitochondrial membrane part	0.000000279	Nil	NP_001006973, NP_071565 *, NP_001008888, NP_001006971.
	Cerebellum	Mitochondrial inner membrane	0.0016	Nil	NP_036702, NP_075581, NP_001006971, NP_037309.

* *P *value by Benjamini correction in DAVID analysis. Other multiple testing correction techniques, Bonferroni and FDR, as provided by DAVID gave the same p values

## Discussion

Using biochemical, histological and proteomic methods, we found that PZH significantly decreased cell death in hippocampus and cerebellum caused by chronic ischemia and hypertensive stroke by possibly preventing brain cells from apoptosis and mitochondria reactive oxygen species (ROS) damage.

We used the ELISA method to detect histone-associated DNA fragments or nucleosomes to study cell death in the hippocampus and the cerebellum two weeks after ischemia in SHR (i.e. ischemia co-existed with hypertension) and in WKY (i.e. ischemia without hypertension). In both WKY and SHR ischemia with PZH treatment groups, the cell deaths were restored to similar levels in the hippocampus as the sham operation groups, whereas the ischemia groups showed significant increases in cell death (Figure 1A). On the other hand, there was no significant difference in the cerebellum between groups (Figure 1B), suggesting that PZH could significantly reduce brain cell death in affected area (e.g. the hippocampus) caused by cerebral ischemia. The protective effects of PZH on ischemia in hippocampus were effective with and without the presence of hypertension. The bilateral CCA were occluded and ischemia would be more severe at regions such as the hippocampus of mid-brain area, while the blood supply to the posterior part of the brain such as cerebellum would be less affected because the vertebral arteries were intact hence the ischemia would be less severe. Therefore, PZH did not show any effect in cerebellum where the less severe ischemia might not have caused significant cell death (Figure 1B). Our choice of hippocampus and cerebellum as the brain regions for study correctly identified two regions with differential damage from the ischemia caused by CCA occlusion.

SHRsp rats with PZH preventive treatment showed significantly decreases in cell deaths as compared to the control group in both hippocampus and cerebellum (Figure 2). Though the stroke in SHRsp is a characteristic clinical feature, the sites of cerebral lesions are somewhat different from those in cerebrovascular diseases; however, the cerebrovascular changes are consistent with those in malignant hypertension [17]. The decreases in cell deaths by PZH at hippocampus and cerebellum caused may be beneficial; however, we also noted that the increases of cell deaths caused by ischemia are only two folds although statistically significant (Figure 1), suggesting that ligation of CCA as a partial and chronic ischemia method may not cause massive cell apoptosis. In addition, we sacrificed the rats two weeks after ligation to examine its chronic effect; any apoptosis caused by the acute effect might have recovered. Nevertheless, two folds of statistically significant increases demonstrated that PZH preventive treatment worked against these increases in apoptosis.

Furthermore, our results of immunostaining were consistent with the cell death assay. Caspase-3 positive cell counts were significantly reduced in the SHR ischemia with PZH treatment as compared to the SHR ischemia control group for both hippocampus and cerebellum (Figure 3). Consistent with cell death assay results; the caspase-3 positive cell numbers were not large, indicating the milder effects of chronic ischemia of ligation of CCA. PZH preventive effects on apoptosis were also statistically significant, suggesting that apoptosis was prevented by PZH in ischemia in both brain regions of SHR.

As to the possible mechanisms of PZH protective effects on ischemia and stroke, proteomic results indicated that the cytochrome b-c_1 _complex subunit 2 (QCR2) was down-regulated in the ischemia with PZH treatment group in both the hippocampus and cerebellum as compared to the ischemia control group (Tables 5 and 6). QCR2 is one of the 11 protein constituents of the ubiquinol:cytochrome c oxidoreductase (bc_1 _complex) [43-45]. The bc_1 _complex as a core of the electronic transfer chains is a component of the eukaryotic respiratory chain in mitochondria catalyzing electron transfer from ubiquinol to cytochrome c, which is coupled to the translocation of protons across the mitochondrial inner membrane from the matrix space [43]. Thus, bc_1 _complex contributes to the electrochemical proton gradient that drives adenosine triphosphate synthesis. A Q-cycle mechanism accounts for the activity of the bc_1 _complex [45]. In the Q-cycle, the semiquinone, an intermediate produced at the Q_0 _site, has a high reactivity with oxygen leading to the production of reactive oxygen species (ROS) which cause damage to the mitochondrial structure and its DNA [45,46]. It has been suggested that this production of ROS may be the major source of damage under some physiological and pathological conditions [46,47]. While QCR2 is not the protein constituent at the Q_0 _site, as a core protein of the complex, the significant down-regulation of QCR2 by PZH may slow down the production of ROS. Our results suggested that PZH minimized the adverse effects of ROS production and oxidative damage which was critical in chronic ischemia and hypertensive stroke despite the fact that ROS production and damage is usually an early event of ischemia insult [46,47]. On the other hand, QCR2 decreased more in cerebellum than in hippocampus (Tables 5 and 6) while cerebellum showed insignificant cell deaths induced by ischemia comparing to hippocampus showed significant cell death (Figure 1). To confirm our findings, future studies on PZH should examine the oxidative status or ROS generation in particular to the role of QCR2.

In the hippocampus, another protein constituent of bc_1 _complex, namely cytochrome bc_1 _complex subunit Rieske (UCRI), was also significantly down-regulated in the PZH group as compared to the control group (Tables 5 and 6). QCR2 and UCRI were placed by DAVID and GOTM with respect to their functional annotations together in the most significant groups (Tables 5 and 6) with other several proteins associated with mitochondrial membrane. Indeed, six out of the nine proteins differentially expressed in hippocampus and five out of the 13 proteins for cerebellum of the PZH and control groups were associated with mitochondria as identified by DAVID (Table 7). For example, the aconitase was up-regulated and prevented oxidative stress [48] and apoptosis [49]. These up- or down-regulated proteins may also play roles in the PZH preventive effects on chronic ischemia and hypertensive stroke. Our results suggested that the target organelle for PZH preventive effects was likely to be the mitochondria and that the target molecule(s) could be the bc_1 _complex or its subunits at least for the hippocampus and cerebellum, which may also be true for the rest of the brain.

One potential limitation of our functional annotation analyses by DAVID and GOTM is the protein lists which are too short from the recommended 100 genes/proteins for avoiding spurious results [40]. We could have increased the protein lists by lowering the factor for creating the differentially expressed protein results from the proteomic experiment; however, considering the variation in 2D proteomic experiment, we think the factor of two was appropriate [39]. Furthermore, to avoid over inference and hence false interpretation, we used only the most significant terms from hippocampus and cerebellum samples by DAVID and GOTM which showed that consistently the mitochondrial membrane was involved and both, QCR2 and UCR1 were among the protein lists. In addition, as mentioned above, six out of the nine proteins for hippocampus and five out of the 13 proteins for cerebellum were functionally annotated with mitochondria by DAVID (Table 7) and similarly by Protein Information Resource (PIR) (Additional file 5). There was a significant association of the PZH preventive treatment and the functions of mitochondria in particular with the bc_1_complex in the aspect of ROS production.

There are the following points worth noting. Firstly, PZH treatment may have independent effects from ischemia on protein expressions and thus the lists of differentially expressed proteins were unrelated with ischemia; however, the composition of PZH [9,24,25] suggests that PZH should have effects on ischemia. Especially the proteins are involved in pathways and organelles for defense against stress, insult or damage. Secondly, the nature of ligation of CCA and cell death and caspase-3 results (Figures 1, 2, 3) suggests that cerebellum would be less affected than hippocampus and would expect a shorter differentially expressed protein list for cerebellum. Our results showed cerebellum had a longer list (Tables 5 and 6). It is difficult to correlate proteomic results with cell death and capase-3 results and in view of the variable 2D electrophoretic results, a shorter protein list for hippocampus than cerebellum may not be interpreted as hippocampus was less affected. This could be due to (1) both protein lists are short and (2) proteomic analysis examines individual protein expressions while cell death and caspase-3 reflect the functions of many proteins together. Therefore, discrepancies between them should not be seen as contradictory or inconclusive.

Mitochondria play a pivotal role in apoptotic pathways [50]. Cytochrome c release triggered by Bax from the mitochondrial membrane initiates apoptosis. Downstream effectors such as caspase-3 are activated leading to apoptosis [51]. The proteomic results did not provide direct evidence as to whether proteins along the apoptotic pathways were up- or down- regulated. However, as the differentially expressed proteins were significantly associated with the mitochondria and their membrane parts, the apoptosis pathway may still be affected. Indeed, our other results showed that cleaved caspase-3 was significantly decreased by PZH preventive treatment in ischemia (Figure 3). Taken together, PZH may affect the electron transfer chain at mitochondria preventing ROS and oxidative damage and maybe the apoptotic pathways, leading to the prevention of cell death due to apoptosis or necrosis as caused by chronic ischemia or hypertensive stroke at hippocampus and cerebellum.

Lastly, while PZH did not prevent the progress of stroke and the subsequent fatality, it significantly delayed the death after stroke had occurred (Tables 3 and 4). This is important in two ways: firstly, we provided no clinical treatment to the rats after stroke had occurred, but this certainly would not be the case for human patients who have suffered a stroke [52]. Therefore, the slightly lesser severity of stroke and significant delay in death would afford a better outcome for human patients. Secondly, stroke in SHRsp rats is imminent, whereas in humans stroke is not a definite outcome, even in hypertensive patients. We may speculate and anticipate that the preventive effects of PZH on human cases of stroke may be very significantly beneficial.

## Conclusion

PZH showed significant preventive effects on limiting the damage or injury caused by ischemia and stroke with and without hypertension in rats. The mechanism of these effects was possibly related to prevention of cell death from apoptosis or ROS and oxidative damage of mitochondria. Further studies to elucidate this mechanism are warranted.

## Abbreviations

PZH: Pien Tze Huang; SHR: spontaneously hypertensive rat; SHRsp: stroke prone SHR; and ELISA: enzyme link immunosorbent assay; ROS: reactive oxygen species; MALDI-TOF: matrix-assisted laser desorption/ionization time-of-flight; CCA: common carotid artery; PBS: phosphate buffered saline; PFA: paraformaldehyde; H_2_O_2_: hydrogen peroxide; 2D: two-dimensional; Vh: voltage hours; MS: mass spectrometric; DAVID: Database for Annotation, Visualization and Integrated Discovery; GOTM: Gene Ontology Tree Machine; SD: standard deviation; S/N: signal to noise

## Competing interests

The authors declare that they have no competing interests.

## Authors' contributions

LZ, MW, MSW, WHK, YTM and DTY designed the study. LZ, WPL, LL, CW, YWW, LHL, HCT and SMN set up and carried out the experiments. LZ, CW, SMN, WHK, YTM and DTY analyzed the results and drafted the manuscript. DTY finalized the manuscript for submission. All authors read and approved the final version of the manuscript.

## Supplementary Material

Additional file 1 **DAVID gene ontology (GO) cluster summary**. in rat hippocampus and cerebellum. Click here for file

Additional file 2 **GOTM gene ontology (GO) cluster summary**. in rat hippocampus and cerebellum. Click here for file

Additional file 3 **Directed acyclic graph (DAG) of proteins**. Expressed proteins in hippocampus. [Note: This graph should be read from top to bottom.] Click here for file

Additional file 4 **Directed acyclic graph of proteins**. Expressed proteins in cerebellum. [Note: This graph should be read from top to bottom.] Click here for file

Additional file 5 **Functional annotation by protein information resources (RIP)**. 3 most frequent gene ontology in hippocampus and cerebellum. Click here for file
