# Supplementary material for: Protective effects and potential mechanisms of Pien Tze Huang on cerebral chronic ischemia and hypertensive stroke
Source: Chin Med. 2010 Oct 18;5:35. doi: 10.1186/1749-8546-5-35 (PMC2984508; doi:10.1186/1749-8546-5-35)
Supplement: Additional file 1 — DAVID gene ontology (GO) cluster summary. in rat hippocampus and cerebellum. [file 1749-8546-5-35-S1.DOC]

DAVID gene ontology (GO) cluster summary with the significant GO terms in each cluster for (A) hippocampus and (B) cerebellum. Clusters were selected if the enrichment score was larger than 1.0, and GO terms with p values less than 0.05 were listed in each cluster. Proteins constituted each cluster and GO term were also listed. * indicated proteins that were up-regulated in the ischemia with PTH treatment group as compared to the ischemia control group.

(A) Hippocampus

| Cluster 1 | Enrichment score: 1.85 | Proteins: NP_036629, NP_112287 *, NP_071565 *, NP_001006973, NP_001006971, NP_001008888, NP_113791, NP_116002, NP_112249. | | |
| --- | --- | --- | --- | --- |
| Category | GO Term | Count | p value | Protein |
| GO component | Mitochondrion | 6 | 0.0000185 | NP_036629, NP_112287 *, NP_071565 *, NP_001006973, NP_001006971, NP_001008888. |
| GO component | Mitochondrial part | 5 | 0.0000212 | NP_112287 *, NP_071565 *, NP_001006973, NP_001006971, NP_001008888. |
| GO component | Mitochondrial respiratory chain | 3 | 0.000155 | NP_001006973, NP_001006971, NP_001008888, |

| Cluster 2 | Enrichment score: 1.00 | Proteins: Q99MZ8, P63039, P32551, P62260, P20788. | | |
| --- | --- | --- | --- | --- |
| Category | GO Term | Count | p value | Protein |
| GO process | Transport | 5 | 0.0418 | NP_116002, NP_071565 *, NP_001006971, NP_113791, NP_001008888. |
| GO process | Establishment of localization | 5 | 0.0472 | NP_116002, NP_071565 *, NP_001006971, NP_113791, NP_001008888. |

(B) Cerebellum

| Cluster 1 | Enrichment score: 3.97 | Proteins: NP_075581, NP_058941 *, NP_036702, NP_001013128 *, NP_037309, NP_059017 *, NP_075412, NP_113971 *, NP_058690, NP_001006971, NP_113808, NP_114039, NP_077374 *, NP_599153. | | |
| --- | --- | --- | --- | --- |
| Category | GO Term | Count | p value | Protein |
| GO function | Binding | 13 | 0.04732 | NP_075581, NP_058941 *, NP_036702, NP_001013128 *, NP_037309, NP_059017 *, NP_075412, NP_113971 *, NP_058690, NP_113808, NP_114039, NP_077374 *, NP_599153. |

| Cluster 2 | Enrichment score: 2.3 | Proteins: NP_075581, NP_001006971, NP_113808, NP_036702, NP_077374 *, NP_037309, NP_113971 *, NP_114039, NP_059017 *, NP_001013128 *. | | |
| --- | --- | --- | --- | --- |
| Category | GO Term | Count | p value | Protein |
| GO component | Mitochondrial inner membrane | 5 | 0.0000317 | NP_075581, NP_001006971, NP_036702, NP_114039, NP_037309. |
| GO component | Organelle inner membrane | 5 | 0.0000395 | NP_075581, NP_001006971, NP_036702, NP_114039, NP_037309. |
| GO component | Mitochondrial membrane | 5 | 0.0000527 | NP_075581, NP_001006971, NP_036702, NP_114039, NP_037309. |

| Cluster 3 | Enrichment score: 1.46 | Proteins: NP_113808, NP_036702, NP_077374 *, NP_037309. | | |
| --- | --- | --- | --- | --- |
| Category | GO Term | Count | p value | Protein |
| GO process | Carboxylic acid metabolic process | 4 | 0.01492 | NP_113808, NP_036702, NP_077374 *, NP_037309. |
| GO process | Organic acid metabolic process | 4 | 0.01507 | NP_113808, NP_036702, NP_077374 *, NP_037309. |
| GO process | Amino acid metabolic process | 3 | 0.03044 | NP_113808, NP_036702, NP_037309. |

| Cluster 4 | Enrichment score: 1.3 | Proteins: NP_075581, NP_077374 *, NP_059017 *, NP_001006971, NP_001013128 *, NP_599153. | | |
| --- | --- | --- | --- | --- |
| Category | GO Term | Count | p value | Protein |
| GO process | Coenzyme metabolic process | 3 | 0.01483 | NP_075581, NP_077374 *, NP_001006971. |
| GO process | Generation of precursor metabolites and energy | 4 | 0.01856 | NP_075581, NP_077374 *, NP_059017 *, NP_001006971. |
| GO process | Cofactor metabolic process | 3 | 0.02106 | NP_075581, NP_077374 *, NP_001006971. |

| Cluster 5 | Enrichment score: 1.22 | Proteins: NP_075581, NP_001006971, NP_001013128 *, NP_036702, NP_077374 *, NP_114039, NP_037309, NP_059017 *, NP_075412, NP_599153, NP_113971 *. | | |
| --- | --- | --- | --- | --- |
| Category | GO Term | Count | p value | Protein |
| GO component | Cytoplasm | 10 | 0.00092 | NP_075581, NP_001006971, NP_001013128 *, NP_036702, NP_077374 *, NP_114039, NP_037309, NP_059017 *, NP_075412, NP_599153. |
| GO component | Intracellular part | 11 | 0.00905 | NP_075581, NP_001006971, NP_001013128 *, NP_036702, NP_077374 *, NP_114039, NP_037309, NP_059017 *, NP_075412, NP_599153, NP_113971 *. |
| GO component | Intracellular | 11 | 0.01977 | NP_075581, NP_001006971, NP_001013128 *, NP_036702, NP_077374 *, NP_114039, NP_037309, NP_059017 *, NP_075412, NP_599153, NP_113971 *. |
