# Supplementary material for: Protective effects and potential mechanisms of Pien Tze Huang on cerebral chronic ischemia and hypertensive stroke
Source: Chin Med. 2010 Oct 18;5:35. doi: 10.1186/1749-8546-5-35 (PMC2984508; doi:10.1186/1749-8546-5-35)
Supplement: Additional file 2 — GOTM gene ontology (GO) cluster summary. in rat hippocampus and cerebellum. [file 1749-8546-5-35-S2.DOC]

GOTM gene ontology (GO) cluster summary according to each GO category, i.e. (A) GO function, (B) GO component and (C) GO process, for hippocampus and cerebellum. Under each GO category, GO terms were listed with p value less than 0.05. * indicated proteins that were up-regulated in the ischemia with PTH treatment group as compared to the ischemia control group.

(A) GO Function

| GO Term | p value | Protein |
| --- | --- | --- |
| Hippocampus | | |
| Protein complex binding | 0.0031 | NP_071565 *, NP_001008888, NP_001006971 |
| Iron-sulfur cluster binding | 0.0165 | NP_001006973, NP_001008888 |
| Metal cluster binding | 0.0165 | NP_001006973, NP_001008888 |
|  |  |  |
| Cerebellum | | |
| Aconitate hydratase activity | 0.000025 | NP_059017 *, NP_077374 * |
| 4 iron, 4 sulfur cluster binding | 0.0038 | NP_059017 *, NP_077374 * |
| Cell surface binding | 0.0053 | NP_599153, NP_075581 |
| Hydro-lyase activity | 0.0265 | NP_059017 *, NP_077374 * |
| Pyridoxal phosphate binding | 0.0371 | NP_599153, NP_037309 |
| Vitamin B6 binding | 0.0424 | NP_599153, NP_037309 |
| Carbon-oxygen lyase activity | 0.0424 | NP_059017 *, NP_077374 * |

(B) GO component

| Term | p value | Protein |
| --- | --- | --- |
| Hippocampus | | |
| mitochondrial membrane part | 0.000000279 | NP_001006973, NP_071565 *, NP_001008888, NP_001006971 |
| mitochondrion | 0.00000055 | NP_001006973, NP_113791, NP_036629, NP_071565 *, NP_112287 *, NP_001008888, NP_001006971 |
| mitochondrial respiratory chain | 0.00000139 | NP_001006973, NP_001008888, NP_001006971 |
|  |  |  |
| Cerebellum | | |
| mitochondrial inner membrane | 0.0016 | NP_036702, NP_075581, NP_001006971, NP_037309 |
| organelle inner membrane | 0.0020 | NP_036702, NP_075581, NP_001006971, NP_037309 |
| mitochondrial membrane | 0.0042 | NP_036702, NP_075581, NP_001006971, NP_037309 |
| mitochondrial envelope | 0.0046 | NP_036702, NP_075581, NP_001006971, NP_037309 |
| mitochondrial part | 0.0138 | NP_036702, NP_075581, NP_001006971, NP_037309 |
| Mitochondrion | 0.0138 | NP_077374 *, NP_036702, NP_075581, NP_001006971, NP_037309 |
| macromolecular complex | 0.023 | NP_113971 *, NP_599153, NP_075581, NP_001006971, NP_037309, NP_058941 *, NP_001013128 *, NP_075412 |

(C) GO process

| Term | p value | Protein |
| --- | --- | --- |
| Hippocampus | | |
| Generation of precursor metabolites and energy | 0.0023 | NP_036629, NP_112287 *, NP_001008888, NP_001006971 |
|  |  |  |
| Cerebellum | | |
| Citrate metabolic process | 0.0011 | NP_059017 *, NP_077374 * |
| Carboxylic acid metabolic process | 0.0068 | NP_113808, NP_059017 *, NP_077374 *, NP_036702, NP_037309 |
| Oxoacid metabolic process | 0.0068 | NP_113808, NP_059017 *, NP_077374 *, NP_036702, NP_037309 |
| Organic acid metabolic process | 0.0069 | NP_113808, NP_059017 *, NP_077374 *, NP_036702, NP_037309 |
| Cellular ketone metabolic process | 0.0074 | NP_113808, NP_059017 *, NP_077374 *, NP_036702, NP_037309 |
| Acetyl-CoA catabolic process | 0.0123 | NP_059017 *, NP_077374 * |
| Coenzyme catabolic process | 0.0123 | NP_059017 *, NP_077374 * |
| Tricarboxylic acid cycle | 0.0123 | NP_059017 *, NP_077374 * |
| Generation of precursor metabolites and energy | 0.0123 | NP_059017 *, NP_077374 *, NP_075581, NP_001006971 |
| Aerobic respiration | 0.0246 | NP_059017 *, NP_077374 * |
| Cofactor catabolic process | 0.0246 | NP_059017 *, NP_077374 * |
| Acetyl-CoA metabolic process | 0.0246 | NP_059017 *, NP_077374 * |
