# Supplementary material for: Protective effects and potential mechanisms of Pien Tze Huang on cerebral chronic ischemia and hypertensive stroke
Source: Chin Med. 2010 Oct 18;5:35. doi: 10.1186/1749-8546-5-35 (PMC2984508; doi:10.1186/1749-8546-5-35)
Supplement: Additional file 3 — Directed acyclic graph (DAG) of proteins. Expressed proteins in hippocampus. [Note: This graph should be read from top to bottom.] [file 1749-8546-5-35-S3.DOC]

Directed acyclic graph (DAG) of the differentially expressed proteins in hippocampus as analyzed by GOTM


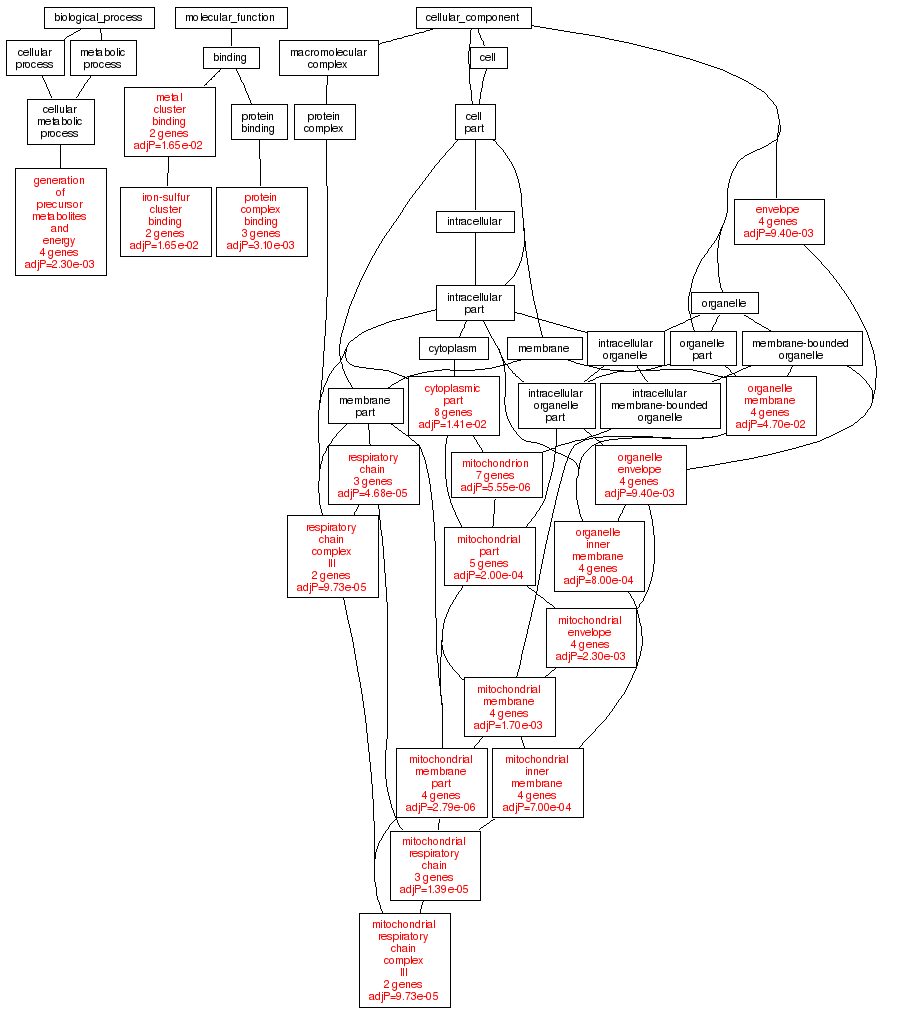


Note: This graph should be read from top to bottom.
