# Supplementary material for: Protective effects and potential mechanisms of Pien Tze Huang on cerebral chronic ischemia and hypertensive stroke
Source: Chin Med. 2010 Oct 18;5:35. doi: 10.1186/1749-8546-5-35 (PMC2984508; doi:10.1186/1749-8546-5-35)
Supplement: Additional file 5 — Functional annotation by protein information resources (RIP). 3 most frequent gene ontology in hippocampus and cerebellum. [file 1749-8546-5-35-S5.DOC]

Summary of functional annotation by Protein Information Resources (PIR) (<http://pir.georgetown.edu/pro/>)

The following table shows the 3 most frequent gene ontology (GO) terms in each GO category for hippocampus and cerebellum. The numbers in parenthesis are the number of proteins included in the term.

| GO Function | | GO Component | | GO Process | |
| --- | --- | --- | --- | --- | --- |
| Hippocampus (9) | Cerebellum (14) | Hippocampus (9) | Cerebellum (14) | Hippocampus (9) | Cerebellum (14) |
|  |  |  |  |  |  |
| Protein binding (8) | Protein binding (11) | Mitochondrion (7) | Mitochondrion (6) | Transport (4) | Regulation of biological process (7) |
| Ion binding (4) | Ion binding (5) | Membrane (6) | Cytoplasm (5) | Regulation of biological process (3) | Transport (6) |
| Nucleotide binding (3) | Catalytic activity (4) | Cytoplasm (4) | Membrane (5) | Electron transport chain (3) | Metabolic process (5) |
